# Genome-Wide Profiling and Analysis of *Arabidopsis* siRNAs

**DOI:** 10.1371/journal.pbio.0050057

**Published:** 2007-02-13

**Authors:** Kristin D Kasschau, Noah Fahlgren, Elisabeth J Chapman, Christopher M Sullivan, Jason S Cumbie, Scott A Givan, James C Carrington

**Affiliations:** 1 Center for Genome Research and Biocomputing, Oregon State University, Corvallis, Oregon, United States of America; 2 Department of Botany and Plant Pathology, Oregon State University, Corvallis, Oregon, United States of America; 3 Molecular and Cellular Biology Graduate Program, Oregon State University, Corvallis, Oregon, United States of America; University of Massachusetts Medical School, United States of America

## Abstract

Eukaryotes contain a diversified set of small RNA-guided pathways that control genes, repeated sequences, and viruses at the transcriptional and posttranscriptional levels. Genome-wide profiles and analyses of small RNAs, particularly the large class of 24-nucleotide (nt) short interfering RNAs (siRNAs), were done for wild-type Arabidopsis thaliana and silencing pathway mutants with defects in three RNA-dependent RNA polymerase (RDR) and four Dicer-like (DCL) genes. The profiling involved direct analysis using a multiplexed, parallel-sequencing strategy. Small RNA-generating loci, especially those producing predominantly 24-nt siRNAs, were found to be highly correlated with repetitive elements across the genome. These were found to be largely RDR2- and DCL3-dependent, although alternative DCL activities were detected on a widespread level in the absence of DCL3. In contrast, no evidence for RDR2-alternative activities was detected. Analysis of RDR2- and DCL3-dependent small RNA accumulation patterns in and around protein-coding genes revealed that upstream gene regulatory sequences systematically lack siRNA-generating activities. Further, expression profiling suggested that relatively few genes, proximal to abundant 24-nt siRNAs, are regulated directly by RDR2- and DCL3-dependent silencing. We conclude that the widespread accumulation patterns for RDR2- and DCL3-dependent siRNAs throughout the *Arabidopsis* genome largely reflect mechanisms to silence highly repeated sequences.

## Author Summary

The discovery of small RNA molecules that inactivate genes during normal growth and development or on invasion by viruses and other mobile genetic elements has profoundly altered our views about how genes are regulated. We set out to investigate the global patterns of small RNA formation and activity across the entire genome of the plant, *Arabidopsis thaliana.* We used new technology and bioinformatics to characterize and compare these patterns in normal plants and in plants with defects in various genes involved in RNA silencing. The results show vast populations of small RNAs in plants and reveal how specialized branches of the RNA-silencing pathway have been adopted for inactivation of different types of genome sequences.

## Abstract 3

There are a lot of small RNAs in *Arabidopis* that regulate other genes. These RNAs are produced by a number of enzymes; the authors have used mutants of these enzymes to see what each of them does.

## Introduction

Most eukaryotes contain RNA-based silencing pathways that function at the transcriptional or posttranscriptional level to negatively regulate or control genes, repetitive sequences, viruses, and mobile elements [1,2]. At the heart of these silencing pathways are small RNAs, which arise from double-stranded RNA (dsRNA) or self-complementary foldback structures by the activity of Dicer or Dicer-like (DCL) ribonucleases [3]. MicroRNAs (miRNAs) derive from RNA polymerase II transcripts that adopt foldback structure, and generally function in trans as negative regulators through base pair interactions with mRNAs [4]. Interaction of miRNAs with target transcripts results in either target degradation or nondegradative inhibition of translation, depending on the degree of sequence complementarity [4]. Trans-acting siRNAs (tasiRNAs) occur in plants and function like miRNAs as posttranscriptional negative regulators of target transcripts, but they form through an RNA-dependent RNA polymerase (RDR)-based mechanism in which precursor transcripts are converted to dsRNA [5,6]. Short interfering RNAs (siRNAs) originating from dsRNA by bidirectional transcription, extended foldbacks with perfect complementarity, or RDR-based mechanisms can also guide transcriptional silencing in which a locus adopts heterochromatic features, including dense cytosine methylation and histone modifications associated with silent chromatin [1,7]. In all forms of RNA silencing, small RNAs associate with effector proteins in the Argonaute (AGO) family [8]. Most AGO proteins contain an RNaseH-like ribonucleolytic domain that is required for RNA-silencing activity [9].

Plants, like other multicellular eukaryotes, have evolved diversified sets of DCL, RDR, AGO, and other factors to provide specialized or preferential functions in RNA-silencing pathways [5,6]. At least four DCL enzymes, in combination with specific dsRNA-binding proteins (including HYL1), catalyze formation of miRNAs or various classes of siRNAs in plants [10–18]. MiRNAs generally require HYL1, which likely works with DCL1 during precursor processing and functions through AGO1 [13,19–22]. For the siRNA pathways, at least three RNA-dependent RNA polymerases (RDR1, RDR2, and RDR6) are functional in plants [17,23–26]. RDR2 works with DCL3 to form chromatin-associated siRNAs (24 nucleotides [nt]) that function through AGO4 [17,27–31]. Functional chromatin-associated siRNAs also require RNA PolIVa and PolIVb, as well as DNA methylation, in a pathway that may include a reinforcement loop [17,24,32–34]. TasiRNAs form through the activity of RDR6, which functions with the precursor-stabilizing factor SGS3 and DCL4 to yield RNAs in a phased 21-nt configuration [35–37]. However, there is clearly diversification of functions or requirements within the tasiRNA class, as subsets of tasiRNAs form by a DRB4-dependent mechanism and function through an AGO7 (ZIPPY)-dependent pathway [38–41]. Additionally, unique combinations of DCL and RDR proteins function during formation of natural antisense siRNA from a locus with bidirectional transcription [42]. Thus, plants use diverse combinations of factors in specialized pathways to silence different classes of endogenous genes and sequences. Further, many of these factors, including DCL2 and DCL4, are also allocated to antiviral silencing pathways involving both cell-autonomous and systemic defense [17,25,43–45].

New technology permits practical deep sequencing of small RNA populations [46,47]. This allows both qualitative discovery of new small RNAs or small RNA classes, as well as quantitative profiling of small RNA populations through direct sequence analysis. In this paper, the small RNA profiles from wild-type (wt) Arabidopsis thaliana and seven RNA-silencing mutants were analyzed. This revealed genome-wide patterns of expression involving each of the major classes of small RNAs, as well as redundancy and nonredundancy among the DCL family.

## Results

### Sequence Analysis of Multiplexed Small RNA Populations

**Figure 1:**
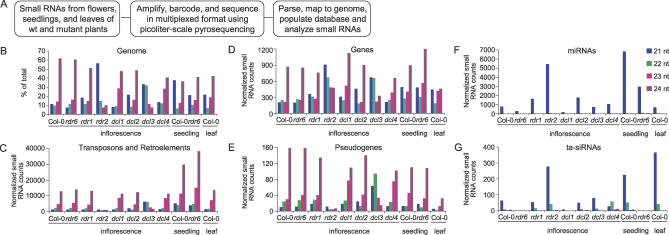
Small RNA Sequencing and the Distribution of Small RNA Loci in Feature Classes (A) Flowchart for high-throughput sequencing and analysis of small RNAs. (B–G) Distribution of small RNAs from wt Col-0 plants and *rdr6–15, rdr1–1, rdr2–1, dcl1–7, dcl2–1, dcl3–1,* and *dcl4–2* mutants in the genome (B), transposons and retroelements (C), genes (D), pseudogenes (E), miRNAs (F), and tasiRNAs (G). In (B), the percentages of small RNAs in each of four size classes within each library are presented. In all other panels, normalized small RNA levels in each feature class are presented.

To determine the extent to which the major small RNA pathways function across the *Arabidopsis* genome, small RNA populations from several tissues of wt and mutant plants were amplified by RT-PCR and sequenced using high-throughput methods (Figure 1A). Amplicons were prepared by 5′ and 3′ adaptor ligation and RT-PCR using small RNA fractions from inflorescence tissue (containing stage 1–12 flowers) of wt Col-0 plants, mutants with defects in each DCL gene *(dcl1–7, dcl2–1, dcl3–1, dcl4–2)* and mutants with defects in each RDR gene for which a function has been established *(rdr1–1, rdr2–1, rdr6–15).* Amplicons from whole seedlings (3-d post-germination) were prepared from Col-0 and *rdr6–15* plants and from leaves of Col-0 plants.

The Col-0 and mutant series samples were sequenced in a multiplexed format in which the 5′ adaptor contained a “barcode” consisting of a 4-nt identifier sequence. Amplicons from three to six samples containing unique barcodes were combined and subjected to picoliter-scale pyrosequencing [47]. Small RNA sequences were parsed from data files and assigned to specific samples through identification of adaptor boundaries and barcode analysis, respectively. Sequences were mapped to the *Arabidopsis* genome by BLAST analysis and deposited into the *Arabidopsis* Small RNA Project (ASRP) database (http://asrp.cgrb.oregonstate.edu/db) [48]. A total of 183,781 unique sequences from 310,906 reads were identified. Given the base-call error rate using this method (~2%), a relatively high proportion of reads contained single-position mismatches relative to an *Arabidopsis* sequence. Positions containing such mismatches were sampled with all possible base substitutions, and the resulting sequences were queried by BLAST analyses. Repairs that led to unambiguous *Arabidopsis* sequences were incorporated in the database, resulting in recovery of 56,654 reads. For the analyses presented here, only sequences that were 20–25 nt in length were used. In total, each wt or mutant population was represented by between 13,688 and 78,583 reads (Table S1). For comparisons involving the mutants, population sizes were normalized using the smallest sequence set (from *dcl2* mutant inflorescence tissue).

In most samples, regardless of tissue type, small RNAs of 24 nt were the predominant size class (Figure 1B). The two exceptions were inflorescence samples from *rdr2* and *dcl3* mutants, in which the 24-nt size class was underrepresented and the 20- to 22-nt size classes were overrepresented. This is consistent with the known role of RDR2 and DCL3 in the biogenesis of 24-nt small RNAs [14,17]. The 24-nt size class was overrepresented in transposon/retroelement and pseudogene feature classes (Figure 1C and 1E). Small RNAs from *MIRNA* genes were predominately 21 nt and accumulated to low levels in the *dcl1* mutant (Figure 1F). Small RNAs from *TAS* genes were predominantly 21 nt and were lost in *dcl1* and *rdr6* mutants (Figure 1G), as expected from the known biogenesis requirements that include miRNA-guided processing of primary transcripts and RDR6-dependent formation of dsRNA [35–37]. The 21-nt tasiRNAs were decreased in *dcl4,* but there was a concomitant increase in alternative tasiRNA size classes due to processing by DCL2 and DCL3 ([11,16]; Figure 1G). The relative abundance of miRNAs and tasiRNAs were elevated in *rdr2* (Figure 1F and 1G). However, given that the 21-nt miRNAs and tasiRNAs do not increase in absolute abundance in the *rdr2* mutant [11,17,46,49], the increase detected here is likely due to overrepresentation in the sequenced population when the abundant 24-nt RNAs are lost. There were relatively few changes detected in the *dcl2* mutant for the feature classes analyzed in detail, although 22-nt small RNAs were lost from some direct and inverted duplication loci (for example, Chromosome III, nt 1961459–1973458; [46]). There were no specific trends detected among small RNA populations in the *rdr1* mutant*.*

### Genome-Wide Distribution of Small RNA-Generating Loci

**Figure 2:**
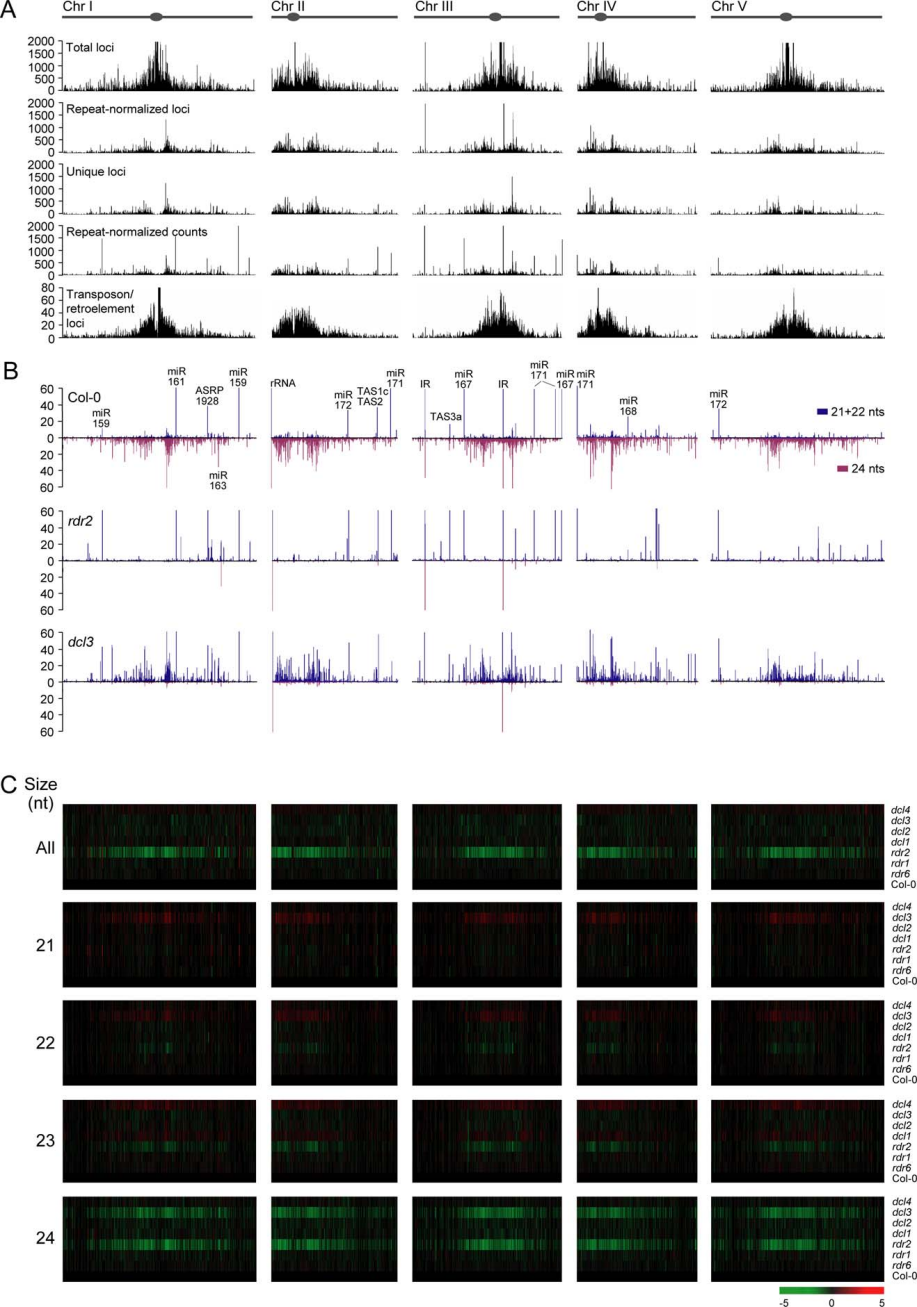
Distribution of Small RNA-Generating Loci from Each Chromosome (A) Scrolling-window analysis (50,000-nt window and 10,000-nt scroll) of small RNA loci. Total, repeat-normalized, and unique small RNA loci, as well as transposon/retroelement loci, are shown. Abundance of repeat-normalized, library-size-normalized counts (individual sequencing reads) are also shown. (B) Scrolling-window analysis of repeat-normalized, library-size-normalized small RNA abundance in Col-0, *rdr2,* and *dcl3.* The summed, 21- and 22-nt size classes (blue, above x-axis) and 24-nt size class (red, below x-axis) were plotted independently. Note that in both (A and B), maximum values plotted were capped at the value corresponding to the maximum y-axis value. (C) Scrolling-window analysis of relative increase (red) or decrease (green) in repeat-normalized, library-size-normalized small RNA abundance in each mutant. Col-0 inflorescence was used as the reference library.

The distribution of small RNA-generating loci from all samples was analyzed across each chromosome. A scrolling-window analysis of all small RNA-homologous sequences (Figure 2A, total loci) was done. These sequences include all loci, regardless of how repetitive, that corresponded to a small RNA. For each chromosome, the density of small RNA-homologous loci was highest in the centromeric and pericentromeric regions (Figure 2). Pericentromeric and centromeric regions contain a high density of repeat sequence classes, such as transposons and retroelements. Indeed, the pericentromeric small RNA locus density roughly corresponded with the density of transposons and retroelements in each chromosome (Figure 2A). This could indicate that the repeat-rich centromeric/pericentromeric regions spawn relatively high numbers of small RNAs, or it could reflect the generation of relatively few small RNAs but from many highly repetitive loci. Three additional scrolling-window analyses were done to distinguish between these possibilities. In the first, only small RNAs from unique loci (Figure 2A, unique loci), to which a small RNA could be unambiguously assigned, were counted. In the second, the value of a prospective small RNA locus was divided by the number of loci corresponding to the small RNA sequence (Figure 2A, repeat-normalized loci). In both of these analyses, the chromosome-wide counts of loci were decreased, although the density was still relatively high in pericentromeric regions. In the third, the abundance of small RNAs in the sequenced populations was plotted after repeat-normalization (Figure 2A, repeat-normalized counts). This resulted in a small RNA-abundance distribution that reflected the repeat-normalized locus density in the pericentromeric regions. This indicates that small RNA-generating loci from pericentromeric regions are abundant, highly active, and diverse. Interestingly, repeat-normalized counts were relatively low at the centromeres, indicating a low small RNA sequence diversity in these regions (Figure 2A). Additionally, sharp isolated peaks of small RNA abundance were detected around the *Arabidopsis* genome. In nearly all cases, these corresponded to 21-nt miRNAs or tasiRNAs, although exceptions included the small RNA population from inverted duplications at two positions in Chromosome III, and the 24-nt miR163 (Figure 2B).

Genome-wide changes in small RNA abundance in each *dcl* and *rdr* mutant (inflorescence tissue) were analyzed by the scrolling-window method and displayed in relation to chromosome position. Data were visualized using heat maps to show over- or underrepresentation of repeat-normalized small RNA levels. For most mutants, small RNA distribution changes were sporadic and localized. In the case of *dcl1,* these localized changes reflected loss of miRNAs and tasiRNAs. In contrast, total small RNAs (20–25 nt) were systematically underrepresented around centromeric and pericentromeric regions of each chromosome in the *rdr2* mutant (Figure 2C). A comparable underrepresentation pattern was not detected in the *dcl3* mutant (Figure 2C), even though these two factors function coordinately in the same pathway. To investigate this discrepancy between *rdr2* and *dcl3* mutants, each small RNA size class was analyzed independently. The 24-nt small RNAs were lost in the same chromosome-wide pattern in both *rdr2* and *dcl3* (Figure 2C), and in a pattern that was coincident with the loss of total small RNAs in *rdr2.* Interestingly, 21- and 22-nt small RNAs were increased in the *dcl3* mutant in a reciprocal chromosome-wide pattern relative to the 24-nt RNA loss pattern (Figure 2B and 2C). A corresponding increase in 21- and 22-nt RNAs was not detected in *rdr2* (Figure 2B and 2C).

The chromosome-wide changes in small RNA profiles were further analyzed by measuring and plotting the abundance of repeat-normalized 21- and 22-nt, as well as 24-nt size class RNAs in Col-0, *rdr2,* and *dcl3.* In Col-0, 24-nt small RNA accumulation correlated well with the distribution of total repeat-normalized small RNA loci, with broad peaks detected in pericentromeric regions (Figure 2B). The abundance of 21- and 22-nt RNAs was characterized by isolated peaks corresponding to miRNAs and tasiRNAs, but also by broad, relatively low peaks that tracked with the 24-nt RNA abundance in the pericentromeric regions (Figure 2B). In the *rdr2* mutant, nearly all 24-nt small RNAs were absent (Figure 2B), although isolated peaks of 24-nt RNAs corresponding to miR163 and a few large inverted duplications were detected. Although the major miRNA and tasiRNA peaks were unaffected in *rdr2,* other 21- and 22-nt small RNAs were generally less abundant from pericentromeric regions (Figure 2B). In *dcl3,* the major redistribution of size classes suggested by the heat maps was clear along each chromosome, particularly in the pericentromeric regions. Except for DCL1-dependent miR163, most 24-nt small RNA peaks and zones were lost and replaced by 21- and 22-nt small RNAs in *dcl3* (Figure 2B). Collectively, these data strongly implicate RDR2 and DCL3 as factors required for widespread biogenesis of 24-nt siRNAs. However, they also reveal genome-wide surrogate DCL functions that act on RDR2-dependent precursors to form 21- and 22-nt small RNAs in the absence of DCL3.

### Effects of *dcl3* and *rdr2* Mutations on Small RNA Populations from Transposons and Retroelements

**Figure 3:**
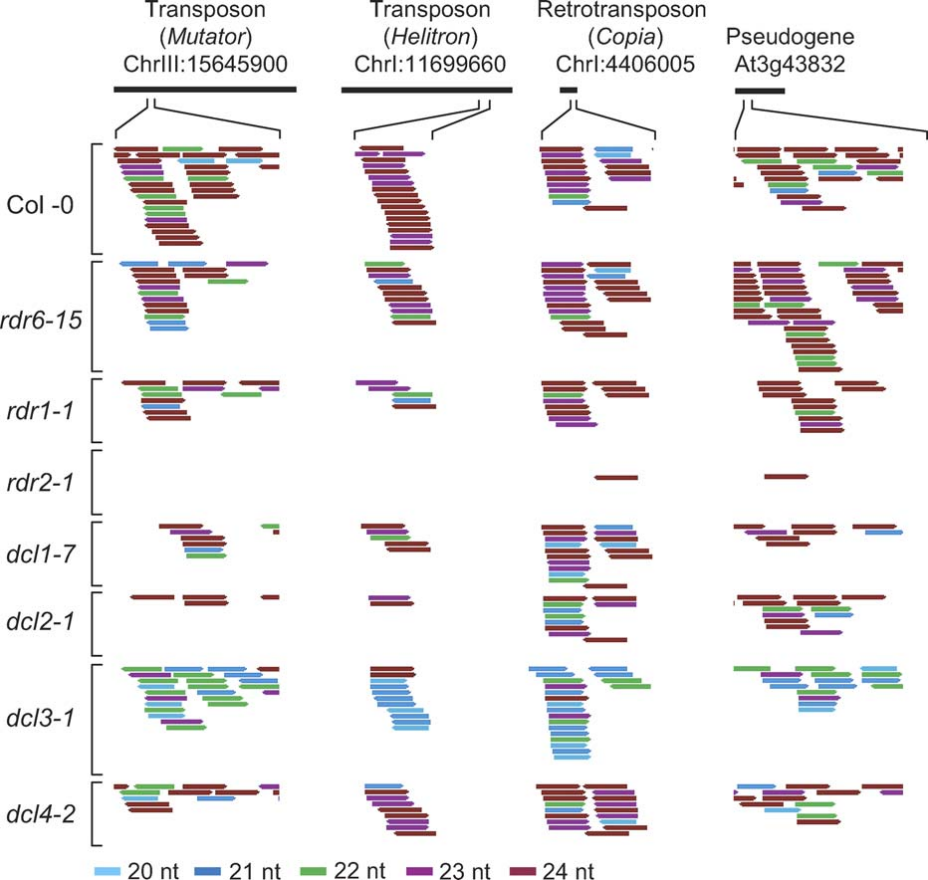
Small RNAs from Segments of Selected Transposons, Retroelements, and Pseudogenes Each unique small RNA is indicated and color-coded based on size.

The small RNA accumulation patterns in and around transposons, retroelements, and pseudogenes were examined in Col-0 and each mutant plant. Small RNAs of all size classes from transposons/retroelements and pseudogenes were decreased specifically in the *rdr2* mutant (Figures 1C, 1E, and 3). In *dcl3,* however, the 24-nt size class was specifically lost and the 21- and 22-nt size classes were elevated from transposons/retroelements and pseudogenes (Figures 1C, 1E, and 3). These siRNAs generally accumulated in bidirectional clusters (Figure 3). This suggests that transposon/retroelements and pseudogenes are targeted by similar 24-nt siRNA-generating systems.

**Figure 4:**
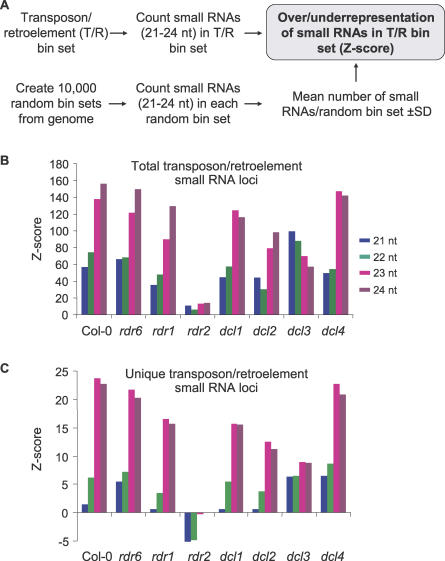
Analysis of Small RNAs Proximal to Transposons and Retroelements (T/R) (A) Method to analyze over- or underrepresentation of small RNAs in T/R bins. (B and C) *Z*-score plots showing overrepresentation (positive) or underrepresentation (negative) of small RNA loci in T/R bins from wt Col-0 and mutant plants. Independent analyses were done for each size class from total (B) and unique (C) small RNA loci.

The association of small RNAs of different size classes in and around transposons and retroelements in wt Col-0 plants was analyzed further. Specifically, the hypothesis that 24-nt, RDR2-dependent small RNAs are overrepresented from loci containing transposons/retroelements was tested. First, all annotated transposons and retroelements [50] were mapped to bins (250 nt) spanning each *Arabidopsis* chromosome (Figure 4A). Each bin overlapped with adjacent bins by 125 nt. A total of 81,328 bins contained transposons or retroelements. For statistical analysis, 10,000 random sets of 81,328 bins were assembled from the genome-wide pool. The randomized bin sets contained 31,860 ± 701 total small RNA loci, with a size distribution similar to that of total small RNAs from Col-0 plants (Figure 1B). Bins containing transposons and retroelements contained 126,311 small RNA loci, indicating a highly significant enrichment (*Z*-score = 135) over the randomized sets (Figure 4B). To normalize for potential bias from highly repeated loci, a similar analysis was done using only single-locus small RNAs that could be assigned unambiguously to one genome position (Figure 4C). Total small RNAs were highly overrepresented (*Z*-score = 20.9) in transposon and retroelement bins using the unique-locus sequences. These analyses were then done for individual 21-, 22-, 23-, and 24-nt size classes. Small RNAs of 23 and 24 nt were most highly overrepresented in the transposon- and retroelement-containing bins using both total (*Z*-scores = 138 and 156, respectively) and unique (*Z*-scores = 23.7 and 22.6, respectively) sequences from Col-0 plants (Figure 4B and 4C). Although 21- and 22-nt small RNAs from transposon and retroelement bins were statistically overrepresented in each analysis using Col-0 data, they were overrepresented to a far lesser extent than were 23- and 24-nt RNAs (Figure 4B and 4C). Further, overrepresentation of all size classes in transposon and retroelement bins was lost or nearly lost in *rdr2* mutant plants (Figure 4B and 4C). In fact, unique 24-nt small RNAs in transposon and retroelement bins were represented at levels similar to those in the random bin sets, and 21- and 22-nt size classes were underrepresented in transposon and retroelement bins in *rdr2* plants (Figure 4C). These data indicate that transposon and retroelement loci are preferentially associated with the 24-nt-generating, RDR2-dependent pathway, but also that transposon- and retroelement-derived small RNAs of all size classes depend on a functional RDR2 protein.

### Small RNA Populations and Clusters in and around Protein-Coding Genes

**Figure 5:**
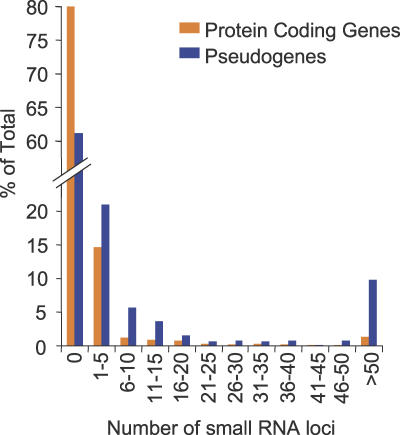
Small RNA Loci in Protein-Coding Genes and Pseudogenes The percentage of genes (orange) and pseudogenes (blue) with 0, 1–5, 6–10, etc., small RNA loci was plotted.

The roles of miRNAs and tasiRNAs in posttranscriptional regulation of target genes in trans are well documented [5,6]. The roles of siRNAs from the RDR2-DCL3 pathway in triggering or maintaining transcriptional silencing of transposon/retroelements and other repeat sequences are also known [51]. However, the extent to which the RDR2-DCL3 pathway regulates expression of functional, protein-coding genes is not understood. Approximately 20% of annotated *Arabidopsis* protein-coding genes (excluding transposons/retroelements) have identity with at least one small RNA sequence represented in the ASRP database. Nearly 75% of these genes spawn five or fewer distinct small RNAs (Figure 5). In contrast, approximately 39% of annotated pseudogenes (excluding transposons/retroelements) have identity to at least one small RNA. For these pseudogenes, the proportion containing abundant arrays of small RNA sequences is much higher than the proportion from protein-coding genes (Figure 5). For both protein-coding genes and pseudogenes, the 24-nt, RDR2-DCL3-dependent class is most abundant (Figure 1D and 1E). However, all small RNA size classes from pseudogenes, but not from protein-coding genes, were negatively impacted by loss of RDR2 (Figure 1D and 1E). The protein-coding gene-derived small RNAs, therefore, are generated by several distinct pathways, including the RDR2-DCL3 pathway.

**Figure 6:**
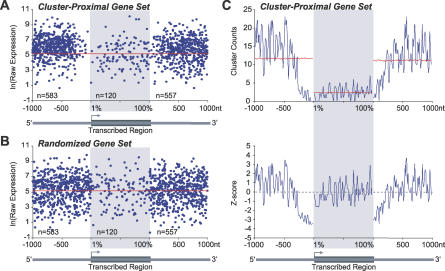
Analysis of Small RNA Clusters in or around Genes (A) Raw expression values in Col-0 inflorescence tissues for genes with clusters up to 1,000 nt upstream (5′) of the transcription start site, within the gene, and 1,000 nt downstream of the 3′ end. Genes are represented as points at positions corresponding to distance from a cluster. (B) Three random gene sets, containing the same numbers of genes *(n)* as in the 5′, 3′, and internal cluster sets, were randomly distributed within the respective zones. Red lines in (A and B) represent the mean expression of 1,000 random sets. (C) Scrolling-window counts of clusters (top), and *Z*-score showing over- or underrepresentation of clusters at positions relative to 5′, internal, and 3′ sites (bottom). Cluster counts and *Z*-score values were determined in 20-nt windows with a 10-nt scroll for observed clusters (blue), and for 1,000 sets of randomly distributed clusters that were averaged at each position (red). Figures below each panel indicate the gene region plotted along the x-axis, with arrows indicating the transcription start sites. Gray boxes mark the intragenic regions plotted on a relative scale (0–100). For each graph, the three independent analyses (5′, 3′, and transcribed regions) were merged in the same plots.

To explore the relationship between small RNAs and protein-coding genes in more detail, gene sets that contain clusters of small RNAs within the transcribed region, or within 1,000 nt of the 5′ or 3′ ends of transcribed regions, were identified. Genes considered in this analysis had both an experimentally determined transcription start site and were represented on the ATH1 expression array (Affymetrix). Small RNA clusters were defined as sets of at least four uni- or bidirectional small RNAs from Col-0 tissue samples, with each small RNA within 200 nt of a neighboring small RNA. After clusters arising from ribosomal RNAs, transfer RNAs, small nuclear RNAs, small nucleolar RNAs, miRNAs, and tasiRNA loci were eliminated, 11,551 clusters were identified. Of these, 8,237 (71%) overlapped with transposon or retrotransposon sequences. Each cluster was reduced to a single point by calculating the mean genome position of first nucleotides of small RNAs. For genes with clusters in the 5′ upstream or 3′ downstream regions, raw gene expression values (inflorescence tissue) were plotted at positions corresponding to distance from cluster means (Figure 6A). For genes with internal clusters, expression values were plotted at points corresponding to the relative position (0–100) of cluster means from the 5′ start site (Figure 6A).

There was no general correlation between cluster proximity and expression values (*p* > 0.30, linear regression) for genes with 5′ upstream or 3′ downstream small RNA clusters (Figure 6A). There was no trend detected to indicate that genes nearer clusters were expressed to levels that differed systematically from those that were more distal to clusters. To determine if expression of any of the three groups of cluster-proximal genes was unique, gene expression in each region was compared (Welch modified two-sample *t*-test) to the means of 1,000 random gene sets composed of the same number of genes. Surprisingly, the median expression level of genes with an upstream cluster was 2.23-fold higher (2.00–2.50, 95% confidence interval) than the median expression level of the random gene sets (Figure 6A and 6B). The median expression level for genes with a downstream cluster was 1.47-fold higher (1.29–1.68, 95% confidence interval) than the median expression value of the random set. In contrast, the gene set with clusters in the transcribed region was not significantly different from genes in the random set (*p* = 0.87, Welch modified two-sample *t*-test).

A scrolling-window count method was used to compare the spatial distribution of clusters in each gene set (5′, 3′, and transcribed cluster sets) to the distribution of clusters in 1,000 positionally randomized sets. Scrolling-window averages for the three gene sets were compared to the mean ± SD of the randomized sets using a *Z*-score statistic. Small RNA clusters were not distributed in a random pattern. Of the 1,204 genes (5.33% of genes on the ATH1 array) with proximity to a cluster, only 8.6% had clusters originating from transcribed sequences, whereas 48.4% and 46.3% had clusters in the 5′ and 3′ regions, respectively (Figure 6A). Importantly, a significant drop-off of small RNA clusters was detected within 200 nt of both 5′ and 3′ gene boundaries (Figure 6A and 6C). Compared to distribution of the randomized control sets (red lines, Figure 6C), clusters in the 5′ and 3′ regions were overrepresented at distal positions and highly underrepresented at proximal positions. Within the transcribed sequences with clusters, the distribution of clusters was relatively similar to the randomly distributed control set (Figure 6C), although there was a slight but significant increase in clusters toward the 3′ end of transcribed sequences (*p* = 0.0006, linear regression; Figure 6C).

**Figure 7:**
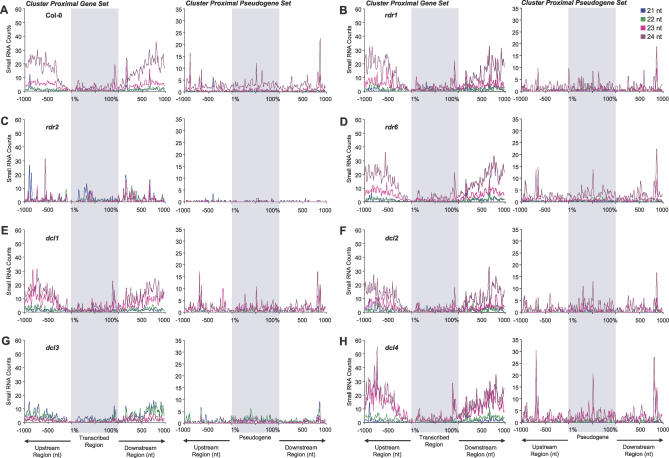
Accumulation of Cluster-Associated Small RNA Size Classes in and around Protein-Coding Genes and Pseudogenes in Col-0 and Mutant Plants Col-0 (A), *rdr1–1* (B), *rdr2–1* (C), *rdr6–15* (D), *dcl1–7* (E), *dcl2–1* (F), *dcl3–1* (G), and *dcl4–2* (H). The scrolling-window method was the same as that presented in Figure 6. The 5′ upstream, 3′ downstream, and transcribed regions were analyzed independently for the cluster-proximal genes. For the cluster-proximal pseudogenes, 5′ upstream, 3′ downstream, and internal pseudogene regions, based on annotation, were analyzed independently. For each graph, the three independent analyses were merged in the same plots.

To determine if the nonrandom distribution patterns of clusters were artifacts of measuring distances to the cluster mean, the distribution of individual small RNA classes in or around genes was analyzed in Col-0 and mutant plants. For comparison, the distribution of small RNAs from cluster-proximal pseudogenes was analyzed in parallel. In both cases, scrolling-window counts of small RNAs in the 21-, 22-, 23-, and 24-nt size classes were made. Like the cluster mean counts in Col-0 plants, the number of small RNAs in the upstream region of genes dropped to near baseline within 200 nt of transcription start sites (Figure 7A). Similarly, the number of small RNAs from sequences in downstream regions proximal to the 3′ ends of genes was relatively low (Figure 7A). In contrast, the number of small RNAs in the upstream and downstream regions near pseudogenes was relatively constant as a function of proximity to the 5′ and 3′ borders (Figure 7A).

As most of the 5′ and 3′ clusters overlap with transposons and retroelements (unpublished data), the majority of small RNAs in Col-0 were in the 24- and 23-nt size classes and were lost in *rdr2* and *dcl3* mutants (Figure 7C and 7G). In both of these mutants, the levels of 21- and 22-nt RNAs in each region were elevated. In the case of *dcl3,* this was due to accumulation of alternative size forms, as shown above (Figures 2 and 3). In the case of *rdr2,* however, this was due primarily to overrepresentation of 21- and 22-nt small RNAs in sequenced populations that were depleted of 23- and 24-nt size class RNAs. Small RNAs in all size classes were lost uniformly within the boundaries, and on the 5′ and 3′ sides, of pseudogenes in *rdr2* (Figure 7C), suggesting that all size classes were RDR2-dependent. In the *dcl3* mutants, pseudogene-derived small RNAs in the 24-nt size class were lost and replaced by 21- and 22-nt RNAs (Figures 1E and 7G).

Among the other mutants, specific peaks of 21-nt small RNAs were lost from flanking and transcribed regions of cluster-proximal genes in *rdr6, dcl1,* and *dcl4* mutants (Figure 7D, 7E, and 7H). Within the transcribed regions, this was due primarily to loss of secondary siRNA formation from miR161 and *TAS2* tasiRNA-targeted transcripts encoding pentatricopeptide repeat proteins (unpublished data). These mutations had relatively few effects on small RNAs from pseudogenes, although isolated 24-nt peaks were elevated in *dcl4* (Figure 7H).

**Figure 8:**
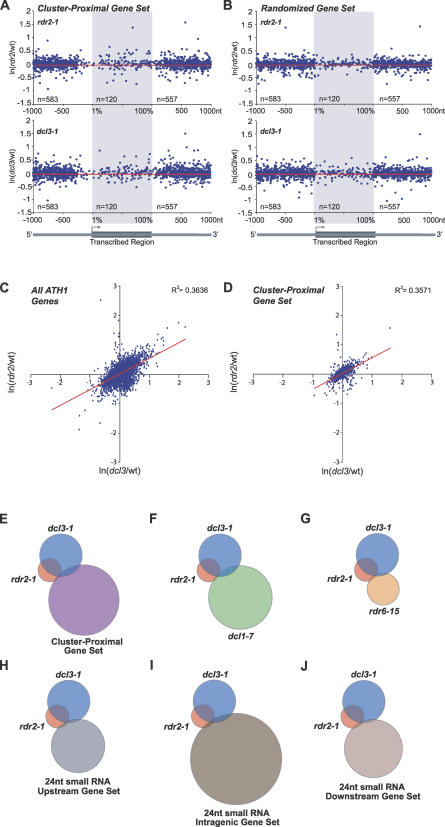
Analysis of the Affect of *rdr2* and *dcl3* on the Expression of Cluster-Proximal Genes (A and B) Fold-change in *rdr2* (upper) and *dcl3* (lower) versus Col-0 inflorescence plotted the same way as in Figure 6A for cluster-proximal genes (A) and the random gene set (B). Red lines in (A and B) represent mean expression of 1,000 random sets. (C and D) Natural log (ln) of fold-change in *dcl3* plotted versus ln of fold-change in *rdr2* for all genes on the ATH1 array (C) or cluster-proximal genes (D). *R* ^2^ is the square of the Pearson correlation and is the percent variation in *rdr2* that is explained by variation in *dcl3.* Red lines are the best-fit lines. (E–J) Venn diagram analysis of genes significantly upregulated at least 1.5-fold (SAM false discovery rate = 0.01) in *rdr2* or *dcl3* and the cluster-proximal gene set (E), *dcl1-*7 (F), *rdr6–15* (G), or all genes with a 24-nt small RNA within 200 nt of the 5′ end (H), within the gene (I), or within 200 nt of the 3′ end (J).

Given that most small RNA clusters near protein-coding genes result from the activity of RDR2 and DCL3, it was reasoned that the expression of cluster-proximal genes might be elevated in *rdr2* and *dcl3* mutants. Elevated expression of cluster-proximal genes in these mutants would suggest that the RDR2-DCL3-dependent small RNA pathway functions constitutively or in a regulated manner to suppress genes. Several analyses were done to test this idea. First, fold-change of expression of each cluster-proximal gene in *rdr2* or *dcl3* was plotted at positions corresponding to distance from cluster means. No significant correlation between fold-change in either *rdr2* or *dcl3* and cluster position was detected for either the cluster-proximal gene set or a random gene set (*p* > 0.3, linear regression) (Figure 8A and 8B). Second, correlation between all genes on the ATH1 array in *rdr2* and *dcl3* was estimated using Pearson's product-moment correlation. The Pearson correlation coefficient for *rdr2* and *dcl3* was *r* = 0.603, indicating that *rdr2* and *dcl3* are positively correlated and that 36% of the variation observed in *rdr2* can be explained by variation in *dcl3* (Figure 8C). This test was then repeated using the cluster-proximal gene set. If cluster-proximal genes were specifically affected by RDR2 and DCL3, it was predicted that the cluster-proximal genes would have a relatively high correlation with genes that were affected by *rdr2* and *dcl3* mutations. However, cluster-proximal genes and all ATH1-represented genes both correlated with *rdr2-* and *dcl3-*affected genes to a similar extent (Figure 8C and 8D). Third, all genes from the ATH1 array were analyzed by the significance analysis for microarrays (SAM) method (false discovery rate = 0.01) [52] to identify genes that were coupregulated by at least 1.5-fold in both *rdr2* and *dcl3.* More than one-half of the 129 genes with elevated expression in *rdr2* were also elevated in *dcl3* (Figure 8E and Table S2), indicating a significant coeffect of the two mutations (*p* = 2.2 × 10^−16^, Fisher exact test). This was in contrast to the limited overlap with gene sets that were upregulated in either *dcl1* or *rdr6* (Figure 8F and 8G). The proportion of cluster-proximal genes that overlapped with either the *rdr2* or *dcl3* upregulated gene sets was not statistically significant (*p* = 0.3, Fisher exact test; Figure 8E). However, of the ten cluster-proximal genes that were significantly upregulated in *rdr2,* seven were also upregulated in *dcl3* (Table S3). These included all four genes that were elevated in *rdr2* with 5′-proximal clusters, and all three genes that were elevated in *rdr2* with 3′-proximal clusters (Table S3).

To more thoroughly examine the effects of *rdr2* and *dcl3* on expression of genes, and to overcome potential biases of examining only cluster-proximal genes, all genes with at least one 24-nt small RNA within 200 nt of the 5′ or 3′ ends were analyzed. In addition, all genes with at least one 24-nt small RNA within the transcribed region were examined. No significant correlation was detected between upregulation of genes in *rdr2* or *dcl3* and the presence of 24-nt small RNAs (*p* > 0.15, Fisher exact test). These gene sets overlapped the *rdr2* and *dcl3* elevated gene sets to a similar, limited extent as did the cluster-proximal gene set (Figure 8H–8J). Therefore, although loss of RDR2 and DCL3 clearly coaffects a set of genes, a few of which were proximal to 24-nt small RNA-generating loci, the overall pattern of small RNA distribution relative to protein-coding genes does not generally reflect a direct influence on gene expression in the tissues analyzed.

## Discussion

### The Application of 454 Sequencing to Small RNA Profiling

High-throughput, parallel-sequencing technology was used to profile small RNA populations in wt plants and mutants with defects in RNA-silencing components. Application of 454 sequencing technology to small RNA profiling is appealing due to the read lengths, throughput, and capacity to multiplex [47]. The latter attribute was particularly useful given the number of samples analyzed in this study. Using a 4-nt molecular barcode on the 5′ adapter, up to six samples were sequenced simultaneously and then resolved computationally. Of course, although multiplexing permits throughput of larger numbers of samples, it is done at the cost of sequencing depth. For analyses that require saturation sequencing or reads for very low-abundance small RNAs, multiplexing would introduce more serious limitations. Further limiting the number of reads in a multiplexed format is the relatively high error rate for base calls [47]. Unambiguous sequence identity was required to parse small RNAs from adapters, map sequences to the *Arabidopsis* genome, and assign sample IDs. The need for three steps to generate a successful read in a multiplexed format, therefore, lowers the yield of usable sequences from a single 454 run.

The value of 454 sequencing as a profiling method arises from the ability to count reads as a quantitative indicator of abundance. Thus, the method works across an open-ended range of values greater than zero and without the need for prior knowledge of any specific sequence. Normalization to adjust for variable sequencing depth between samples was achieved using a linear proportion method. However, interpretation of representational differences in abundance of specific small RNAs or small RNA classes in different samples required careful interpretation. For example, increased representation of miRNAs in sequenced populations from the *rdr2* mutant, as shown here and documented previously [49,53], was due to miRNA overrepresentation in a low-diversity pool of sequences that lack most 24-nt siRNAs. The absolute abundance of miRNAs in the *rdr2* mutants is relatively unaffected [11,17,46,49]. In contrast, increased representation of 21-nt small RNAs from transposons and retroelements in the *dcl3* mutant was due to absolute increases in abundance of this size class.

### Utilization and Molecular Redundancy of the RDR2-DCL3-Dependent Small RNA Biogenesis Pathway

By far, the most abundant and broadly utilized small RNA pathway in *Arabidopsis* depends on RDR2 and DCL3, resulting in accumulation of a highly diverse population of primarily 24-nt RNAs. Using a variety of measures, this pathway has a major affiliation with repeated sequences, including transposons and retroelements. The 24-nt RNAs are highly overrepresented from these feature classes, even when quantitative adjustments for repeat density are used (Figure 4). In other words, the repeated sequences analyzed here have disproportionately high activity as loci that spawn 24-nt small RNAs. Analysis of the unique sequence component of transposon/retroelement small RNA populations, as well as the repeat-normalized small RNA data from transposon/retroelement sequences, allows us to rule out the possibility that the high number of small RNAs from these feature classes is due simply to locus abundance.

In the *rdr2* mutant, all size classes of small RNAs from transposons, retroelements, and pseudogenes were lost, suggesting that other RDR proteins (RDR1 and RDR6) are largely unable to compensate for loss of RDR2. This was in striking contrast to the *dcl3* mutant, in which only the 24-nt size class was lost. In fact, there was a major redistribution of sizes from 24 nt to 21/22 nt at repeat loci in the *dcl3* mutant. This almost certainly indicates that alternate DCLs that yield 21- and 22-nt small RNAs gain considerably more access to RDR2-dependent dsRNA in the absence of DCL3. DCL1 and DCL4 are known to catalyze formation of predominantly 21-nt RNAs [11,13,15,16,18], while DCL2 catalyzes formation of 22-nt RNAs [11,17,43]. We suggest that RDR2-dependent dsRNA has the potential to be processed by multiple DCLs, although preferentially by DCL3, in wt plants. Are these alternate-size small RNAs formed in *dcl3* mutant plants functional to guide events (DNA methylation and chromatin modifications) associated with transcriptional silencing? Functional analyses of transcriptional silencing at several loci, including *AtSN1* retroelements and the repeat sequences in the *FWA* gene promoter, reveal that *dcl3* mutants are generally weaker than *rdr2* mutants [17,54,55]. Partial redundancy or compensation in the absence of DCL3 may explain the limited transcriptional silencing in *dcl3* mutants.

Why might DCL3, but not RDR2, be subject to molecular redundancy or surrogacy? The events that route transcripts and small RNAs through the RDR2-DCL3-dependent pathway were found to be spatially localized in nuclear compartments [14,56]. PolIVa, which is necessary for most small RNAs generated by RDR2-DCL3 [24,32–34,57,58], is spatially dispersed in the nucleus [14]. RDR2, DCL3, AGO4, PolIVb, and siRNAs, on the other hand, accumulate in RNA-processing centers (Cajal bodies) associated with the nucleolus [14,56]. It is likely, therefore, that RNA-trafficking mechanisms deliver RDR2 substrates to processing centers. Access to alternative RDR proteins (RDR1 and RDR6) may be prevented by distinct accumulation patterns outside of these centers, or by unique association of substrate transcripts with factors that interact specifically with RDR2. Although each of the DCL proteins have nuclear transport signals, only DCL3 is known to accumulate in processing centers [14,17,59,60]. We suggest that there is relatively free access to RDR2-dependent dsRNA by alternate DCLs when DCL3 is absent. DsRNA synthesis, siRNA duplex formation, and AGO4 loading with siRNAs likely occurs in a spatially restricted complex [14,56], in which functional DCL3 may occupy a preferential position (and thus normally exclude other DCLs) through unique associations with other factors.

### Role of RDR2-DCL3-Dependent Small RNA Pathway in Regulation of Genes and Genetic Buffering

The RDR2-DCL3-dependent pathway is necessary to maintain transcriptional silencing of some (though not all) transposons and repeat sequences [17,46,54,55,61]. Loss of this pathway in *Arabidopsis* is associated with accumulation of defects over multiple generations [11]. Specific transposons in maize are progressively reactivated in the absence of MOP1, the ortholog of *Arabidopsis* RDR2 [62–66]. The patterns of siRNA accumulation across the *Arabidopsis* genome strongly support the concept that the RDR2-DCL3 pathway provides maintenance or generational reinforcement of transposon and repeat-sequence silencing.

While some of the developmental defects that accumulate in the absence of the RDR2-DCL3 pathway [11] may be due to mutagenesis associated with transposon activity, some may involve stochastic, epigenetic events that release silent chromatin to an active state through histone modifications and loss of DNA methylation. In these cases, the RDR2-DCL3-dependent siRNA pathway may have a role in initiation and reinforcement of epigenetic patterns, particularly at silenced loci that depend on non-CG methylation [17,54,67,68]. Loss of RDR2 and DCL3, therefore, may affect some transcriptionally silent loci in unpredictable ways, or may have no effect at all, depending on the extent to which maintenance of epigenetic marks requires continuous siRNA formation.

The extent to which small RNA-based transcriptional silencing affects spatial-temporal gene regulation during growth and development appears to be somewhat different than what might be predicted based on the abundant small RNA accumulation patterns. Several genes, such as *FWA,* are known to be maintained in a silent state through chromatin silencing that is triggered by siRNAs from direct repeats in the promoter and initial transcribed sequences [54,55]. We also note the results of Chan et al. [69] indicate that loss chromatin-associated RNA-silencing factors results in developmental abnormalities. Further, loss of RDR2 and DCL3 results in coordinate, though relatively modest, affects across hundreds of *Arabidopsis* genes (Figure 8C). However, no general correlation was found between proximity to abundant 24-nt RNA clusters and impact of *rdr2* and *dcl3* mutations on expression level (Figure 8). Although a small set of cluster-proximal genes were significantly up in both *rdr2* and *dcl3* mutant plants, as would be expected if siRNAs functioned constitutively or transiently to suppress gene expression, we detected no general trend for the cluster-proximal genes. The results of expression profiling of cluster-proximal genes suggest that relatively few of these genes are directly affected by the clusters (Figure 8).

Interestingly and counterintuitively, genes with 5′- or 3′-proximal, but not internal, clusters had higher overall expression levels than would be expected by random chance (Figure 6). While this could suggest that small RNAs in 5′ or 3′ regions directly enhance the expression of nearby genes, the results of expression-profiling experiments using *rdr2* and *dcl3* mutants argue against this idea (Figure 8). The basis for higher-than-expected expression may be due, however, to indirect consequences of transcription of the small RNA-generating loci. In fact, the abundant, 24-nt RNA-generating loci may be transcribed relatively efficiently in a significant proportion of cases, thus yielding high levels of substrate for dsRNA synthesis by RDR2. How this would influence access to promoters in adjacent genes remains to be determined experimentally.

A case can be made that evolution favors the avoidance of small RNA-generating loci near regulatory sequences of expressed genes. A paucity of small RNA clusters is clearly evident near the core promoter and upstream regions within 200 nt of the 5′ start sites of protein-coding genes, but not around pseudogenes. Interestingly, cytosine methylation patterns around *Arabidopsis* protein-coding genes, but not pseudogenes or transposons, were shown recently to be underrepresented around core promoter and 3′ flanking regions [70,71]. Zilberman et al. [71] suggest that the absence of DNA methylation in these regions has evolved to define the initiation and termination sites for active genes in complex genomes. In the Drosophila melanogaster genome, these regions are also sites of active nucleosome exchange and RNA polymerase occupation [72]. We suggest that promoters and termination sites have evolved to limit small RNA biogenesis activity that could potentially trigger DNA methylation and repressive chromatin, which would interfere with entry and exit points for RNA polymerase.

Given that the distribution of small RNA clusters around genes is roughly indicative of the distribution of transposons and retroelements, we favor the hypothesis that transposons and retroelements have significantly influenced the structure of the expressed component of plant genomes. In fact, the introgression of transposon and retroelement sequences within expressed genes is known to influence epigenetic regulation [55,61,73,74]. Further, the consequences of proliferation of such sequences within the *Arabidopsis* genome are known to influence broad chromatin domains, such as the heterochromatic knob region on Chromosome IV [55]. Invasion of genes by transposons and retroelements, therefore, is likely to be suppressive and detrimental in most cases. For expanding gene families, however, introgression of these sequences may also preserve genetic information in a silent state [75]. This would result in maintenance of potentially useful genetic information or variation while suppressing problems associated with elevated gene dosage. Previously, we proposed that posttranscriptional silencing mechanisms operate on an evolutionary scale to buffer the effects of rapidly expanding gene families [76]. Aberrant recombination events that yield inverted duplications may form miRNA-like negative regulators, which would suppress closely related family members, preserve genetic information in a silent state, and absorb gene-dosage effects. Thus, plants may use a variety of RNA-based silencing mechanisms to both suppress invasive elements and tame the effects of rapid gene expansion.

## Materials and Methods

### Plant materials.

A. thaliana (Col-0 background) mutants containing *dcl1–7, dcl2–1, dcl3–1, dcl4–2, rdr1–1, rdr2–1,* and *rdr6–15* alleles were described previously [15,17,35–37]. Plants used for inflorescence tissue (containing stage 0–12 flowers) were grown under standard greenhouse conditions with supplemental light on a 16-h photoperiod. Tissue was collected 6 h into a light cycle. For seedling tissue, seeds were washed with 80% ethanol/0.1% Triton X-100 for 10 min, then 30% Clorox bleach/0.1% Triton X-100 for 10 min, and rinsed three times with sterile water. Seeds were imbibed and cold-treated for 72 h in 0.1% agar prior to plating on MS agar medium overlaid with nylon mesh (Nitex 03–100/47; Sefar America, http://www.sefar.us). Agar plates were oriented vertically in a growth chamber with a 16-h photoperiod. 3-d-old seedlings were collected 4–6 h into a light cycle.

### Small RNA amplification and sequencing.

Small RNA libraries were constructed as described previously [77], with the following modifications. The sequence of the 3′ adaptor was altered (5′ATTGATGGTGCCTACA3′), and the 5′ adaptor was replaced with a chimeric RNA/DNA oligonucleotide [78]. Sequences within the RNA portion of the 5′ adaptor were varied to generate eight unique 5′ adaptors (1–1, ATCGTAG**GCAC**CUGAUA; 1–2, ATCGTAG**GCCA**CUGAUA; 1–3, ATCGTAG**GCUG**CUGAUA; 1–4, ATCGTAG**GCGU**CUGAUA; 2–1, ATCGTAG**CGAC**CUGAUA; 2–2, ATCGTAG**CGCA**CUGAUA; 2–3, ATCGTAG**CGUG**CUGAUA; 2–4, ATCGTAG**CGGU**CUGAUA). Throughout small RNA isolation and 3′-adaptor ligation steps, RNA was purified using 17% denaturing PAGE with trace amounts of ^32^P-radiolabeled RNA transcripts as internal size standards [78]. Reverse transcription was primed using a primer complementary to the 3′ adaptor (RT primer; [78]) and cDNA was amplified by PCR using ExTaq DNA Polymerase (Takara, http://www.takara-bio.com) and 3′ PCR FusionB (5′- GCCTTGCCAGCCCGCTCAGATTGATGGTGCCTACAG-3′) and 5′ PCR FusionA (5′-GCCTCCCTCGCGCCATCAGATCGTAGGCACCTGATA3′) primers. Variant 1–1 of 5′ PCR FusionA is shown; underlined bases were varied to match the 5′ adaptor used during RNA ligation steps. PCR primers contained the “A” and “B” tag sequences used by 454 Life Sciences (http://www.454.com) during sample processing and sequencing [47]. DNA amplicons were gel-purified using 4% Metaphor agarose and isolated using a QIAEX II Gel Extraction Kit (Qiagen, http://www1.qiagen.com) followed by phenol and chloroform extractions and ethanol precipitation. DNA amplicons from three to six source libraries were pooled and sequenced by 454 Life Sciences.

### Small RNA parsing and analysis.

FASTA formatted files containing 706,567 reads from four 454 Life Sciences sequencing runs were parsed by Perl scripts using the barcoded 5′ and 3′ adapter sequences to identify the small RNA/adapter boundaries. Parsed small RNA sequences were associated to the correct source library, mapped to the *Arabidopsis* genome (TAIR version 6) by BLAST analysis, and deposited in a MySQL database. Sequences with single nucleotide mismatches were tested by substituting all other nucleotides at the mismatch position, followed by BLAST against the *Arabidopsis* genome. Those sequences that could be unambiguously repaired with a single nucleotide substitution were added to the database with an appropriate annotation. *Arabidopsis* small RNA sequence data were added to the ASRP database (http://asrp.cgrb.oregonstate.edu/db) [48]. The number of reads for each inflorescence source library was 78,583 (Col-0), 19,761 *(rdr1–1),* 19,626 *(rdr2–1),* 46,892 *(rdr6–15),* 17,839 *(dcl1–7),* 13,688 *(dcl2–1),* 30,318 *(dcl3–1),* and 29,497*(dcl4–2).* The number of reads for the two seedling libraries was 22,462 (Col-0) and 23,980 *(rdr6–15).* The single-leaf library contained 15,826 (Col-0) reads. For all comparisons between mutants, small RNA counts were normalized to the library containing the fewest reads for each tissue type.

A second MySQL database was created using a “bin” format in which each chromosome was divided into 250-nt segments containing all corresponding annotation and small RNA data. These data were used to analyze chromosome-wide distribution of small RNA loci and small RNA abundance. Repeat-normalization was done by dividing each small RNA-homologous locus count by the total number of loci with identity to that small RNA. For data shown in Figure 2, locus distribution plots were generated by summing numbers of all or repeat-normalized small RNA loci in 200-bin (50,000 nt) windows with a scroll of 20 bins (10,000 nt) for consecutive data points. For each library, the total, read-normalized isolations were calculated in each window, the data were normalized to Col-0, and the natural log was plotted on heat maps using R v2.3.0 [79].

Database annotation for all protein-coding genes was from version 6 of the *Arabidopsis* genome annotation (http://www.arabidopsis.org). Genes annotated as transposons and retrotransposons, pseudogenes, miRNA-, and tasiRNA-generated genes, and all structural RNA genes were analyzed independently from protein-coding genes. The pseudogene set consisted of the 648 pseudogenes that did not overlap with transposons and retroelements. Transposon and retroelement sequences and annotation were from RepBase v10.0.1 [50].

Small RNA clusters were identified using data only from Col-0 inflorescence and seedling libraries. A cluster was defined to contain a minimum of four small RNAs, each separated from nearest neighbors by a maximum of 200 nt.

### Microarray and statistical analyses.

All microarray data used are available at Gene Expression Omnibus (GEO) [80,81]. Col-0, *dcl3–1,* and *rdr2–1* were from experiments previously described in Allen et al. [35]. Col-0 (control for *rdr6–15*), *rdr6–15,* Col-0 (control for *dcl1–7*), and *dcl1–7* were from experiments previously described in Xie et al. [16].

Random gene sets were generated by randomly selecting genes from the ATH1 array. Linear regression analyses, Welch modified two-sample *t*-tests, Fisher exact tests, and Pearson's product-moment correlation tests were done using R v2.3.0 [79]. SAM analyses were done using the SAM plugin for Microsoft Excel v2.23A [52]. All SAM analyses used a FDR of 0.01 and a post-analysis fold-change cutoff of 1.5. Venn diagram analyses were done using the venn package (v1.5) for the statistical programming package, R (http://www.stats.uwo.ca/faculty/murdoch/software).

## Supporting Information

Table S1 Small RNA Sequences Parsed from Each Library (27 KB DOC)

Table S2 Significantly Upregulated Genes in *rdr2* and *dcl3* Mutants (19 KB DOC)

Table S3 Significantly Upregulated Small RNA Cluster Proximal Genes in *rdr2* and *dcl3*
(61 KB DOC)

### Accession Numbers

The Gene Expression Omnibus (http://www.ncbi.nlm.nih.gov/geo) accession number for the small RNA sequences discussed in this paper is GSE6682. GEO accession numbers for the microarray data are GSE2473 (samples GSM47020, GSM47021, GSM47022, GSM47031, GSM47032, GSM47033, GSM47046, GSM47047, and GSM47048), and GSE3011 (samples GSM65929, GSM65930, GSM65931, GSM65935, GSM65936, GSM65937, GSM65938, GSM65939, GSM65940, GSM65941, GSM65942, and GSM65943) [16,35].

## Abbreviations

AGO: Argonaute
DCL: Dicer-like
dsRNA: double-stranded RNA
miRNA: microRNA
nt: nucleotide
RDR: RNA-dependent RNA polymerase
SAM: significance analysis for microarrays
siRNA: short interfering RNA
tasiRNA: trans-acting siRNA
wt: wild-type
