# Supplementary material for: Genome-Wide Profiling and Analysis of Arabidopsis siRNAs
Source: PLoS Biol. 2007 Feb 13;5(3):e57. doi: 10.1371/journal.pbio.0050057 (PMC1820830; doi:10.1371/journal.pbio.0050057)
Supplement: Table S2 — (19 KB DOC) [file pbio.0050057.st002.doc]

Table S2. Significantly upregulated genes in *rdr2 and dcl3* mutants

Gene	Affected Genotype[a]	Fold Change[b]	Gene	Affected Genotype[a]	Fold Change[b]	Gene	Affected Genotype[a]	Fold Change[b]
At3g28540	dcl3	1.500	At3g56090	dcl3	1.535	At2g15880	dcl3	1.564
At5g44170	dcl3	1.501	At1g14330	dcl3	1.535	At5g10830	dcl3	1.567
At5g54300	dcl3	1.503	At1g74010	dcl3	1.535	At2g39210	dcl3	1.567
At1g51820	dcl3	1.503	At4g10040	dcl3	1.538	At3g22240	dcl3	1.567
At1g25450	dcl3	1.503	At1g72510	dcl3	1.540	At3g05910	dcl3	1.568
At2g40460	dcl3	1.504	At1g61290	dcl3	1.541	At1g76160	dcl3	1.569
At3g51300	dcl3	1.505	At1g60240	dcl3	1.541	At3g44735	dcl3	1.569
At3g20520	dcl3	1.505	At1g72180	dcl3	1.541	At3g21420	dcl3	1.570
At4g01870	dcl3	1.506	At5g02200	dcl3	1.543	At5g18270	dcl3	1.572
At4g12420	dcl3	1.507	At5g46770	dcl3	1.543	At5g45840	dcl3	1.573
At2g04030	dcl3	1.508	At1g23100	dcl3	1.543	At3g26960	dcl3	1.573
At4g26690	dcl3	1.508	At3g19020	dcl3	1.544	At4g35060	dcl3	1.576
At3g20865	dcl3	1.508	At1g12070	dcl3	1.546	At1g76090	dcl3	1.580
At1g44100	dcl3	1.509	At2g01630	dcl3	1.547	At1g30700	dcl3	1.580
At2g34340	dcl3	1.511	At2g33570	dcl3	1.547	At2g31945	dcl3	1.584
At3g25165	dcl3	1.511	At1g64200	dcl3	1.547	At2g38480	dcl3	1.585
At3g54240	dcl3	1.511	At5g21105	dcl3	1.548	At5g13200	dcl3	1.586
At3g57260	dcl3	1.511	At4g03400	dcl3	1.549	At3g46660	dcl3	1.587
At5g54610	dcl3	1.512	At1g10550	dcl3	1.549	At3g06420	dcl3	1.589
At3g25170	dcl3	1.513	At5g44300	dcl3	1.549	At1g23350	dcl3	1.590
At2g44130	dcl3	1.514	At1g04250	dcl3	1.550	At5g25450	dcl3	1.591
At3g20220	dcl3	1.514	At3g01530	dcl3	1.551	At3g59350	dcl3	1.591
At2g46360	dcl3	1.514	At5g61820	dcl3	1.552	At1g15670	dcl3	1.591
At1g13890	dcl3	1.514	At4g38540	dcl3	1.552	At2g46860	dcl3	1.595
At4g13560	dcl3	1.515	At4g30270	dcl3	1.552	At2g43290	dcl3	1.595
At1g52540	dcl3	1.516	At2g19800	dcl3	1.553	At1g04430	dcl3	1.598
At2g13350	dcl3	1.517	At5g61010	dcl3	1.554	At5g57760	dcl3	1.599
At4g18800	dcl3	1.517	At3g05400	dcl3	1.556	At3g02910	dcl3	1.599
At4g17460	dcl3	1.517	At2g38640	dcl3	1.556	At5g16500	dcl3	1.601
At5g02490	dcl3	1.519	At5g50790	dcl3	1.556	At5g27980	dcl3	1.601
At5g07110	dcl3	1.521	At1g69570	dcl3	1.557	At1g59660	dcl3	1.602
At3g52600	dcl3	1.521	At1g28430	dcl3	1.557	At1g14360	dcl3	1.602
At5g18150	dcl3	1.523	At5g48470	dcl3	1.558	At3g08970	dcl3	1.604
At3g06740	dcl3	1.525	At2g21490	dcl3	1.558	At5g19240	dcl3	1.604
At1g62300	dcl3	1.527	At2g01850	dcl3	1.559	At5g25340	dcl3	1.604
At4g18425	dcl3	1.527	At4g18050	dcl3	1.559	At1g62180	dcl3	1.605
At1g23480	dcl3	1.529	At4g39670	dcl3	1.560	At5g02580	dcl3	1.605
At5g07440	dcl3	1.534	At2g30010	dcl3	1.561	At4g04955	dcl3	1.606
At4g30610	dcl3	1.534	At1g15520	dcl3	1.563	At5g24010	dcl3	1.607
At5g10695	dcl3	1.534	At2g23810	dcl3	1.564	At4g16563	dcl3	1.608

Gene	Affected Genotype[a]	Fold Change[b]	Gene	Affected Genotype[a]	Fold Change[b]	Gene	Affected Genotype[a]	Fold Change[b]
At4g27580	dcl3	1.608	At1g59730	dcl3	1.654	At3g49270	dcl3	1.711
At2g42580	dcl3	1.608	At5g36720	dcl3	1.656	At2g19770	dcl3	1.713
At2g34770	dcl3	1.608	At5g50260	dcl3	1.658	At2g17500	dcl3	1.714
At1g65310	dcl3	1.608	At1g63090	dcl3	1.660	At1g70090	dcl3	1.714
At1g21520	dcl3	1.609	At4g04490	dcl3	1.662	At5g38710	dcl3	1.715
At4g34138	dcl3	1.609	At1g53885	dcl3	1.662	At1g49800	dcl3	1.719
At5g05440	dcl3	1.610	At1g36370	dcl3	1.663	At4g28780	dcl3	1.719
At4g15550	dcl3	1.611	At4g38400	dcl3	1.663	At4g29285	dcl3	1.724
At4g01700	dcl3	1.614	At4g37370	dcl3	1.663	At1g08920	dcl3	1.724
At5g39050	dcl3	1.614	At1g51250	dcl3	1.663	At3g61930	dcl3	1.724
At2g27500	dcl3	1.615	At2g02010	dcl3	1.663	At1g72430	dcl3	1.725
At5g58782	dcl3	1.616	At4g08850	dcl3	1.665	At5g24655	dcl3	1.726
At2g37470	dcl3	1.619	At1g78600	dcl3	1.665	At3g07470	dcl3	1.728
At3g62640	dcl3	1.619	At1g28270	dcl3	1.667	At2g31880	dcl3	1.729
At5g05600	dcl3	1.620	At5g39280	dcl3	1.668	At4g02380	dcl3	1.729
At2g39680	dcl3	1.621	At3g08860	dcl3	1.670	At1g72930	dcl3	1.731
At2g30610	dcl3	1.623	At1g26820	dcl3	1.671	At3g26980	dcl3	1.733
At3g58620	dcl3	1.624	At1g68610	dcl3	1.673	At1g01310	dcl3	1.733
At4g30140	dcl3	1.630	At4g25590	dcl3	1.673	At1g19380	dcl3	1.736
At5g27870	dcl3	1.631	At5g55050	dcl3	1.673	At1g70985	dcl3	1.738
At4g20860	dcl3	1.632	At2g36410	dcl3	1.675	At5g13190	dcl3	1.742
At5g24030	dcl3	1.633	At1g13340	dcl3	1.678	At4g15800	dcl3	1.743
At3g26860	dcl3	1.634	At4g20260	dcl3	1.688	At5g52640	dcl3	1.745
At1g35490	dcl3	1.634	At2g40670	dcl3	1.688	At5g56540	dcl3	1.749
At5g40780	dcl3	1.634	At1g64405	dcl3	1.688	At3g15540	dcl3	1.751
At5g54095	dcl3	1.635	At3g44320	dcl3	1.692	At1g32170	dcl3	1.751
At1g74820	dcl3	1.636	At4g37800	dcl3	1.694	At5g50200	dcl3	1.752
At1g04770	dcl3	1.637	At3g26970	dcl3	1.694	At3g05490	dcl3	1.753
At4g34750	dcl3	1.639	At4g13340	dcl3	1.694	At2g02990	dcl3	1.755
At4g12720	dcl3	1.643	At5g52360	dcl3	1.695	At1g72150	dcl3	1.756
At1g63260	dcl3	1.643	At1g77760	dcl3	1.695	At3g01250	dcl3	1.756
At3g57450	dcl3	1.644	At4g18950	dcl3	1.695	At5g48570	dcl3	1.761
At3g19550	dcl3	1.646	At2g19000	dcl3	1.696	At1g17990	dcl3	1.766
At1g24170	dcl3	1.647	At5g17480	dcl3	1.700	At4g15190	dcl3	1.766
At2g02140	dcl3	1.649	At2g02810	dcl3	1.700	At1g28130	dcl3	1.770
At1g61720	dcl3	1.650	At5g16590	dcl3	1.702	At5g61605	dcl3	1.770
At1g19890	dcl3	1.650	At3g50560	dcl3	1.703	At5g54380	dcl3	1.772
At5g15350	dcl3	1.651	At4g14365	dcl3	1.708	At3g03430	dcl3	1.772
At2g47550	dcl3	1.651	At1g67865	dcl3	1.708	At3g28340	dcl3	1.779
At1g09240	dcl3	1.652	At4g28490	dcl3	1.709	At4g31000	dcl3	1.782
At4g22870	dcl3	1.652	At5g61790	dcl3	1.711	At5g65730	dcl3	1.783

Gene	Affected Genotype[a]	Fold Change[b]	Gene	Affected Genotype[a]	Fold Change[b]	Gene	Affected Genotype[a]	Fold Change[b]
At4g20830	dcl3	1.784	At4g15610	dcl3	1.903	At2g21640	dcl3	2.214
At2g30140	dcl3	1.785	At4g29520	dcl3	1.910	At5g57520	dcl3	2.224
At2g45210	dcl3	1.790	At3g56200	dcl3	1.913	At1g24996	dcl3	2.233
At2g31730	dcl3	1.792	At2g18660	dcl3	1.913	At5g53250	dcl3	2.242
At1g47280	dcl3	1.797	At5g42800	dcl3	1.918	At1g65490	dcl3	2.263
At5g64510	dcl3	1.798	At4g04610	dcl3	1.924	At1g34040	dcl3	2.286
At3g13950	dcl3	1.804	At3g01290	dcl3	1.930	At3g30775	dcl3	2.300
At4g12580	dcl3	1.807	At1g19500	dcl3	1.937	At4g08950	dcl3	2.300
At2g36950	dcl3	1.811	At3g55890	dcl3	1.942	At1g03870	dcl3	2.318
At3g51660	dcl3	1.813	At5g07990	dcl3	1.953	At5g52070	dcl3	2.321
At4g26780	dcl3	1.819	At5g23020	dcl3	1.955	At3g04000	dcl3	2.364
At4g33420	dcl3	1.821	At2g38310	dcl3	1.956	At1g61800	dcl3	2.372
At5g63850	dcl3	1.822	At3g11340	dcl3	1.960	At3g24510	dcl3	2.375
At2g45180	dcl3	1.823	At2g38900	dcl3	1.970	At3g28210	dcl3	2.396
At4g03210	dcl3	1.825	At2g17230	dcl3	1.973	At3g16040	dcl3	2.419
At2g37770	dcl3	1.826	At5g39610	dcl3	1.974	At4g21990	dcl3	2.422
At2g43820	dcl3	1.827	At3g22600	dcl3	1.976	At5g40690	dcl3	2.437
At3g61020	dcl3	1.829	At3g52620	dcl3	1.978	At2g21220	dcl3	2.498
At1g69840	dcl3	1.830	At1g05680	dcl3	1.988	At4g19430	dcl3	2.500
At5g26060	dcl3	1.832	At3g50480	dcl3	1.994	At3g45970	dcl3	2.520
At4g18290	dcl3	1.833	At3g10890	dcl3	2.001	At5g47350	dcl3	2.533
At2g30990	dcl3	1.833	At1g65500	dcl3	2.013	At2g29470	dcl3	2.570
At5g05340	dcl3	1.833	At5g62730	dcl3	2.013	At5g48850	dcl3	2.614
At1g59710	dcl3	1.834	At1g14870	dcl3	2.017	At3g49580	dcl3	2.653
At4g09030	dcl3	1.839	At4g21200	dcl3	2.021	At1g76960	dcl3	2.702
At4g18010	dcl3	1.844	At1g57990	dcl3	2.027	At2g32210	dcl3	2.704
At1g50460	dcl3	1.852	At5g40155	dcl3	2.029	At3g23730	dcl3	2.775
At1g70810	dcl3	1.853	At3g07340	dcl3	2.044	At2g45220	dcl3	2.796
At5g44020	dcl3	1.854	At1g30040	dcl3	2.054	At3g04210	dcl3	2.868
At1g75870	dcl3	1.856	At4g35725	dcl3	2.055	At1g08930	dcl3	2.915
At5g27350	dcl3	1.858	At4g37990	dcl3	2.085	At5g64120	dcl3	2.937
At4g30280	dcl3	1.860	At2g29460	dcl3	2.100	At4g15750	dcl3	2.978
At4g02130	dcl3	1.865	At4g37150	dcl3	2.113	At2g30930	dcl3	2.983
At5g38760	dcl3	1.868	At3g12580	dcl3	2.134	At1g02930	dcl3	2.985
At1g37130	dcl3	1.869	At5g11360	dcl3	2.135	At2g43510	dcl3	3.023
At5g37770	dcl3	1.873	At3g59900	dcl3	2.142	At2g41100	dcl3	3.105
At3g04070	dcl3	1.879	At3g60980	dcl3	2.151	At4g25810	dcl3	3.143
At3g48580	dcl3	1.883	At2g41230	dcl3	2.189	At5g26220	dcl3	3.165
At3g12110	dcl3	1.887	At5g01740	dcl3	2.201	At5g24660	dcl3	3.193
At5g65390	dcl3	1.893	At5g06720	dcl3	2.205	At2g44460	dcl3	3.447
At1g16950	dcl3	1.894	At4g18395	dcl3	2.209	At3g22231	dcl3	3.623

Gene	Affected Genotype[a]	Fold Change[b]	Gene	Affected Genotype[a]	Fold Change[b]	Gene	Affected Genotype[a]	Fold Change[b]
At2g29350	dcl3	4.403	At1g67860	rdr2	1.695	At1g66390	rdr2/dcl3	1.710/1.830
At5g44130	dcl3	4.454	At2g17000	rdr2	1.708	At5g17450	rdr2/dcl3	1.750/1.794
At2g04460	rdr2	1.502	At5g22300	rdr2	1.738	At5g63790	rdr2/dcl3	1.750/2.038
At1g79620	rdr2	1.505	At3g05890	rdr2	1.802	At4g36110	rdr2/dcl3	1.760/1.824
At5g41400	rdr2	1.506	At3g48970	rdr2	1.804	At1g52830	rdr2/dcl3	1.767/2.114
At4g00050	rdr2	1.511	At2g36700	rdr2	1.831	At2g32190	rdr2/dcl3	1.773/2.723
At1g33560	rdr2	1.512	At1g64360	rdr2	1.838	At2g21200	rdr2/dcl3	1.782/1.653
At5g26330	rdr2	1.513	At3g54120	rdr2	1.862	At1g72110	rdr2/dcl3	1.801/1.522
At4g24120	rdr2	1.516	At4g16640	rdr2	1.923	At4g00330	rdr2/dcl3	1.811/1.670
At3g26450	rdr2	1.520	At1g65480	rdr2	1.979	At3g28740	rdr2/dcl3	1.812/2.833
At4g00440	rdr2	1.525	At1g02220	rdr2	1.980	At5g03120	rdr2/dcl3	1.813/2.140
At5g33290	rdr2	1.527	At3g30140	rdr2	2.032	At1g64640	rdr2/dcl3	1.837/1.595
At3g53230	rdr2	1.527	At1g61920	rdr2	2.107	At4g08300	rdr2/dcl3	1.841/1.800
At5g10430	rdr2	1.530	At5g19880	rdr2	2.259	At4g12730	rdr2/dcl3	1.846/2.015
At1g62480	rdr2	1.531	At5g36930	rdr2	2.304	At5g35735	rdr2/dcl3	1.855/1.965
At1g35910	rdr2	1.532	At3g07130	rdr2	2.498	At3g05900	rdr2/dcl3	1.864/1.792
At5g36940	rdr2	1.536	At2g13770	rdr2	3.463	At3g03840	rdr2/dcl3	1.869/1.673
At3g56290	rdr2	1.545	At2g19850	rdr2	3.498	At4g34135	rdr2/dcl3	1.884/1.978
At1g62790	rdr2	1.551	At5g35490	rdr2	3.589	At5g59515	rdr2/dcl3	1.885/1.978
At5g26655	rdr2	1.559	At2g03080	rdr2	3.958	At5g37600	rdr2/dcl3	1.888/1.802
At1g28570	rdr2	1.559	At3g30720	rdr2	12.672	At3g50770	rdr2/dcl3	1.895/2.191
At3g25290	rdr2	1.560	At2g34070	rdr2/dcl3	1.509/1.789	At4g14130	rdr2/dcl3	1.904/2.697
At4g33050	rdr2	1.588	At3g17390	rdr2/dcl3	1.516/1.710	At1g09970	rdr2/dcl3	1.906/2.242
At1g35730	rdr2	1.593	At5g58650	rdr2/dcl3	1.521/1.572	At1g75750	rdr2/dcl3	1.929/3.403
At1g29450	rdr2	1.599	At2g37540	rdr2/dcl3	1.525/1.665	At1g21550	rdr2/dcl3	1.952/1.691
At4g30190	rdr2	1.599	At1g22590	rdr2/dcl3	1.530/1.794	At2g36790	rdr2/dcl3	1.956/2.526
At4g10580	rdr2	1.605	At1g14130	rdr2/dcl3	1.546/1.536	At4g38860	rdr2/dcl3	1.996/1.849
At1g75780	rdr2	1.608	At3g50900	rdr2/dcl3	1.561/1.572	At5g49480	rdr2/dcl3	2.005/2.325
At1g72900	rdr2	1.612	At1g28600	rdr2/dcl3	1.563/1.712	At3g49780	rdr2/dcl3	2.021/2.026
At2g31900	rdr2	1.618	At2g41730	rdr2/dcl3	1.568/1.846	At1g43910	rdr2/dcl3	2.029/2.955
At4g39840	rdr2	1.624	At5g09440	rdr2/dcl3	1.568/1.946	At1g22530	rdr2/dcl3	2.042/2.230
At1g03820	rdr2	1.634	At2g21650	rdr2/dcl3	1.577/1.695	At1g11545	rdr2/dcl3	2.067/2.277
At5g42860	rdr2	1.643	At4g05590	rdr2/dcl3	1.621/1.726	At1g75040	rdr2/dcl3	2.078/1.947
At3g14660	rdr2	1.652	At3g13790	rdr2/dcl3	1.636/1.614	At1g05560	rdr2/dcl3	2.090/2.582
At1g28230	rdr2	1.653	At1g12110	rdr2/dcl3	1.645/1.619	At5g60490	rdr2/dcl3	2.101/1.649
At5g18060	rdr2	1.662	At3g22370	rdr2/dcl3	1.652/1.879	At1g70270	rdr2/dcl3	2.158/3.913
At1g12180	rdr2	1.662	At5g26340	rdr2/dcl3	1.656/1.806	At4g13180	rdr2/dcl3	2.169/2.391
At1g35290	rdr2	1.666	At3g29810	rdr2/dcl3	1.690/1.848	At3g22840	rdr2/dcl3	2.204/1.765
At3g44450	rdr2	1.677	At1g76520	rdr2/dcl3	1.695/1.918	At1g76690	rdr2/dcl3	2.286/2.525
At2g13760	rdr2	1.685	At2g41380	rdr2/dcl3	1.700/2.440	At3g16150	rdr2/dcl3	2.389/2.225
At1g63020	rdr2	1.688	At4g22710	rdr2/dcl3	1.710/1.532	At2g29420	rdr2/dcl3	2.415/3.000

Gene	Affected Genotype[a]	Fold Change[b]		Gene	Affected Genotype[a]	Fold Change[b]		Gene	Affected Genotype[a]	Fold Change[b]
At4g31870	rdr2/dcl3	2.511/2.046		At5g42180	rdr2/dcl3	2.701/2.620		At1g19020	rdr2/dcl3	3.540/6.780
At4g14690	rdr2/dcl3	2.601/1.854		At1g68620	rdr2/dcl3	3.099/3.918		At5g45890	rdr2/dcl3	5.237/6.919
At4g04830	rdr2/dcl3	2.601/2.845		At1g29660	rdr2/dcl3	3.364/3.302				

[a]Significantly affected in *rdr2*, *dcl3*, or *rdr2* and *dcl3* (*rdr2*/*dcl3*) (FDR = 0.01).

[b]Fold change of *rdr2* and/or *dcl3* versus wt Col-0.
