# Supplementary material for: Genome-Wide Profiling and Analysis of Arabidopsis siRNAs
Source: PLoS Biol. 2007 Feb 13;5(3):e57. doi: 10.1371/journal.pbio.0050057 (PMC1820830; doi:10.1371/journal.pbio.0050057)
Supplement: Table S3 — (61 KB DOC) [file pbio.0050057.st003.doc]

| Table S3. Significantly upregulated small RNA cluster proximal genes in *rdr2* and *dcl3* | | | | |
| --- | --- | --- | --- | --- |
| Accession | Cluster Location^a^ | Affected Genotype^b^ | Fold Change^c^ | Gene Family^d^ |
| At2g04030 | Upstream | *dcl3* | 1.51 | Heat shock protein |
| At2g23810 | Upstream | *dcl3* | 1.56 | TETRASPANIN |
| At2g43290 | Upstream | *dcl3* | 1.60 | Calmodulin-like protein |
| At5g24030 | Upstream | *dcl3* | 1.63 | C4-dicarboxylate transporter/Malic acid transport protein |
| At3g49270 | Upstream | *dcl3* | 1.71 | Unknown |
| At4g09030 | Upstream | *dcl3* | 1.84 | Arabinogalactan-protein |
| At5g07990 | Upstream | *dcl3* | 1.95 | Flavonoid 3'-hydroxylase/Cytochrome P450 |
| At3g17390 | Upstream | *rdr2*/*dcl3* | 1.52/1.71 | S-adenosylmethionine synthetase |
| At3g29810 | Upstream | *rdr2*/*dcl3* | 1.69/1.85 | Phytochelatin synthetase |
| At3g50770 | Upstream | *rdr2*/*dcl3* | 1.90/2.19 | Calmodulin-related protein |
| At1g09970 | Upstream | *rdr2*/*dcl3* | 1.91/2.24 | Leucine-rich repeat transmembrane protein kinase |
| At1g15670 | Gene | *dcl3* | 1.59 | Kelch repeat-containing F-box protein |
| At1g47280 | Gene | *dcl3* | 1.80 | Unknown |
| At3g50480 | Gene | *dcl3* | 1.99 | RPW8 R-gene family protein |
| At5g52070 | Gene | *dcl3* | 2.32 | Agenet domain-containing protein |
| At3g26450 | Gene | *rdr2* | 1.52 | Major latex protein-related |
| At1g28230 | Gene | *rdr2* | 1.65 | Purine permease |
| At5g35490 | Gene | *rdr2* | 3.59 | Unknown |
| At5g44170 | Downstream | *dcl3* | 1.50 | Unknown |
| At2g30010 | Downstream | *dcl3* | 1.56 | Unknown |
| At5g13200 | Downstream | *dcl3* | 1.59 | GRAM domain-containing protein / ABA-responsive protein-related |
| At4g04955 | Downstream | *dcl3* | 1.61 | Allantoinase |
| At5g39050 | Downstream | *dcl3* | 1.61 | Transferase |
| At1g28270 | Downstream | *dcl3* | 1.67 | Rapid alkalinization factor protein |
| At5g24655 | Downstream | *dcl3* | 1.73 | Unknown |
| At1g72930 | Downstream | *dcl3* | 1.73 | Toll-Interleukin-Resistance domain-containing protein |
| At2g18660 | Downstream | *dcl3* | 1.91 | Expansin protein |
| At3g28210 | Downstream | *dcl3* | 2.40 | Zinc finger (AN1-like) protein |
| At3g22231 | Downstream | *dcl3* | 3.62 | Pathogen and circadian controlled |
| At2g34070 | Downstream | *rdr2/dcl3* | 1.51/1.79 | Unknown |
| At2g41380 | Downstream | *rdr2/dcl3* | 1.70/2.44 | Embryo-abundant protein-related |
| At2g32190 | Downstream | *rdr2/dcl3* | 1.77/2.72 | Unknown |
| ^a^Upstream is within 1,000 nt of the transcription start site; Gene is within the genic region; Downstream is within 1,000 nt of the end of the transcript. | | | | |
| ^b^Significantly affected in *rdr2*, *dcl3*, or *rdr2* and *dcl3* (*rdr2*/*dcl3*) (FDR = 0.01). | | | | |
| ^c^Fold change versus wt Col-0 (*rdr2*/*dcl3*). | | | | |
| ^d^TAIR annotation | | | | |
