# Supplementary material for: Genome-Wide Profiling and Analysis of Arabidopsis siRNAs
Source: PLoS Biol. 2007 Feb 13;5(3):e57. doi: 10.1371/journal.pbio.0050057 (PMC1820830; doi:10.1371/journal.pbio.0050057)
Supplement: Supplementary file 3 [file pbio.0050057.st001.doc]

Table S1. Small RNA sequences parsed from each library

Plant	Tissue	15-35 nt	20-25 nt		
		Reads[a]	Reads	Genomic hit[b]	Unique[c]
Col-0	inf	119606	92550	78583	57966
Col-0	leaf	44112	20977	15826	8113
Col-0	sdlg	37293	27546	22462	12718
rdr6-15	inf	64020	53031	46892	37774
rdr6-15	sdlg	55955	33513	23980	16223
rdr1-1	inf	27186	22568	19761	14755
rdr2-1	inf	45507	25163	19626	6249
dcl1-7	inf	31088	22040	17839	14692
dcl2-1	inf	11067	8879	7824	10405
dcl3-1	inf	22933	15844	13509	22951
dcl4-2	inf	17343	12925	11264	22448

[a]sequences parsed from adapters
[b]sequences with a minimum of one perfect genomic locus
[c]number of unique sequences identified in each library
